# Co-Occurrence Patterns of Common and Rare Leaf-Litter Frogs, Epiphytic Ferns and Dung Beetles across a Gradient of Human Disturbance

**DOI:** 10.1371/journal.pone.0038922

**Published:** 2012-06-11

**Authors:** Johan A. Oldekop, Anthony J. Bebbington, Nathan K. Truelove, Niklas Tysklind, Santiago Villamarín, Richard F. Preziosi

**Affiliations:** 1 Faculty of Life Sciences, The University of Manchester, Manchester, United Kingdom; 2 Institute of Development Policy and Management, The University of Manchester, Manchester, United Kingdom; 3 Graduate School of Geography, Clark University, Worcester, Massachusetts, United States of America; 4 College of Natural Sciences, Bangor University, Bangor, United Kingdom; 5 Museo Ecuatoriano de Ciencias Naturales, Quito, Ecuador; Monash University, Australia

## Abstract

Indicator taxa are commonly used to identify priority areas for conservation or to measure biological responses to environmental change. Despite their widespread use, there is no general consensus about the ability of indicator taxa to predict wider trends in biodiversity. Many studies have focused on large-scale patterns of species co-occurrence to identify areas of high biodiversity, threat or endemism, but there is much less information about patterns of species co-occurrence at local scales. In this study, we assess fine-scale co-occurrence patterns of three indicator taxa (epiphytic ferns, leaf litter frogs and dung beetles) across a remotely sensed gradient of human disturbance in the Ecuadorian Amazon. We measure the relative contribution of rare and common species to patterns of total richness in each taxon and determine the ability of common and rare species to act as surrogate measures of human disturbance and each other. We find that the species richness of indicator taxa changed across the human disturbance gradient but that the response differed among taxa, and between rare and common species. Although we find several patterns of co-occurrence, these patterns differed between common and rare species. Despite showing complex patterns of species co-occurrence, our results suggest that species or taxa can act as reliable indicators of each other but that this relationship must be established and not assumed.

## Introduction

The accelerating decline of ecosystems and the loss of biodiversity is forcing conservation scientists to develop quick, cost-effective and accurate tools to measure biological responses to environmental changes [1]–[3]. Although surrogate species or indicator taxa are commonly implemented as ecological monitoring tools, there is no consensus about their ability to act as proxies of biological patterns. Large and rare charismatic organisms, for example, often fail to accurately represent rapid environmental changes and are frequently unable to provide information about regional ecological trends [4]. Similarly, the occurrence of rare or threatened species does not necessarily overlap with areas of high biodiversity [5]–[7].

While there are numerous studies reporting no consistent relationships between indicator groups [5], [8], [9], there are as many publications supporting the use of surrogate taxa [10]–[12] as well as a further category of studies reporting mixed results [13]–[15]. The lack of consensus about the use of biodiversity surrogate measures can be attributed to differing methodologies and geographical scales as well as differences between geographical regions and/or biomes [16], [17]. Complementarity approaches, for example, which use surrogacy to select series of sites that collectively maximise species richness, outperform surrogate methods that select areas maximising species richness alone [16]. Similarly, approaches that assume surrogacy based on extrapolated data, tend to perform better than measures based on field data, yet run the risk of reporting false positives, leading to potentially wrong decision making when selecting areas for conservation [17].

Since the Convention on Biological Diversity’s target to “achieve by 2010 a significant reduction of the current loss of biodiversity” the drive has been to devise methods to monitor biodiversity trends at the global scale or over large geographical regions [2], [18], [19]. Consequently, many studies have focused on measuring the co-occurrence of rare or threatened species using large geographical areas as units of assessment [20]. While such coarse-resolution studies can provide useful information for global policy decisions [19], they often show larger levels of species co-occurrence than fine-resolution studies, which are often conducted at local scales [21]. Although human-driven land-cover change is the leading cause of environmental degradation [22], coarse-resolution studies have often failed to assess the effect of human disturbance on patterns of species co-occurrence [13], [23]. Furthermore, coarse-scale studies often fail to contribute useful information for local management decisions, which often rely on monitoring results within locally managed areas.

While species rarity is often used to weight conservation priorities [24], the exclusion of occasional species, can substantially increase estimates of conservation value in tropical forests [25]. In recent years, several fine-resolution studies have focused on how measures of species diversity change across human disturbance gradients. Gregory et al. [26], for example, use a pan-European dataset to show how generalist farmland bird populations have decreased in relation to increases in agricultural intensification. More recently, Barlow et al. [27], Pardini et al. [28] and Kessler et al. [29] use large multi-taxon assessments to measure responses to anthropogenic impacts in tropical forests. While these studies contribute to our understanding of the ability of human modified landscapes to harbour species diversity, there is little information about the impact of environmental degradation on common and rare species and whether impacts affect the relative contributions of common and rare species to general patterns of species diversity [5], [23], [30]. Differentiating the effects of environmental degradation on the co-occurrence of total, common and rare species will help elucidate the intricacies of anthropogenic impacts on species richness patterns, and contribute to the development of better monitoring protocols to measure impacts of environmental changes as well as help the evaluation of conservation potentials of human modified landscapes.

Here, we contribute to the current understanding of how environmental degradation affects species richness patterns by examining how human disturbance and forest degradation affect the co-occurrence of common and rare species of epiphytic ferns, leaf litter frogs and dung beetles in a tropical forest environment in the Ecuadorian Amazon. We concentrate on these taxa because they have been taxonomically well studied in Ecuador [31], [32] have been successfully used to assess environmental degradation in the neotropics [27], [28], [33], and have simpler sampling protocols and/or more stringent habitat requirements than other commonly used indicators (e.g. birds and mammals see [27]). More specifically, we measure the response of total, common and rare species to environmental degradation and assess the relative contribution of rare and common species to the patterns of total richness in each taxon. We then analyse their potential as monitoring tools by assessing the ability of common and rare species to act as surrogate measures of each other. Because rare species are often more speciose but less relatively abundant than common species [34], our reasoning is that environmental degradation will differentially impact common and rare species.

## Materials and Methods

### Study Site

**Figure 1:**
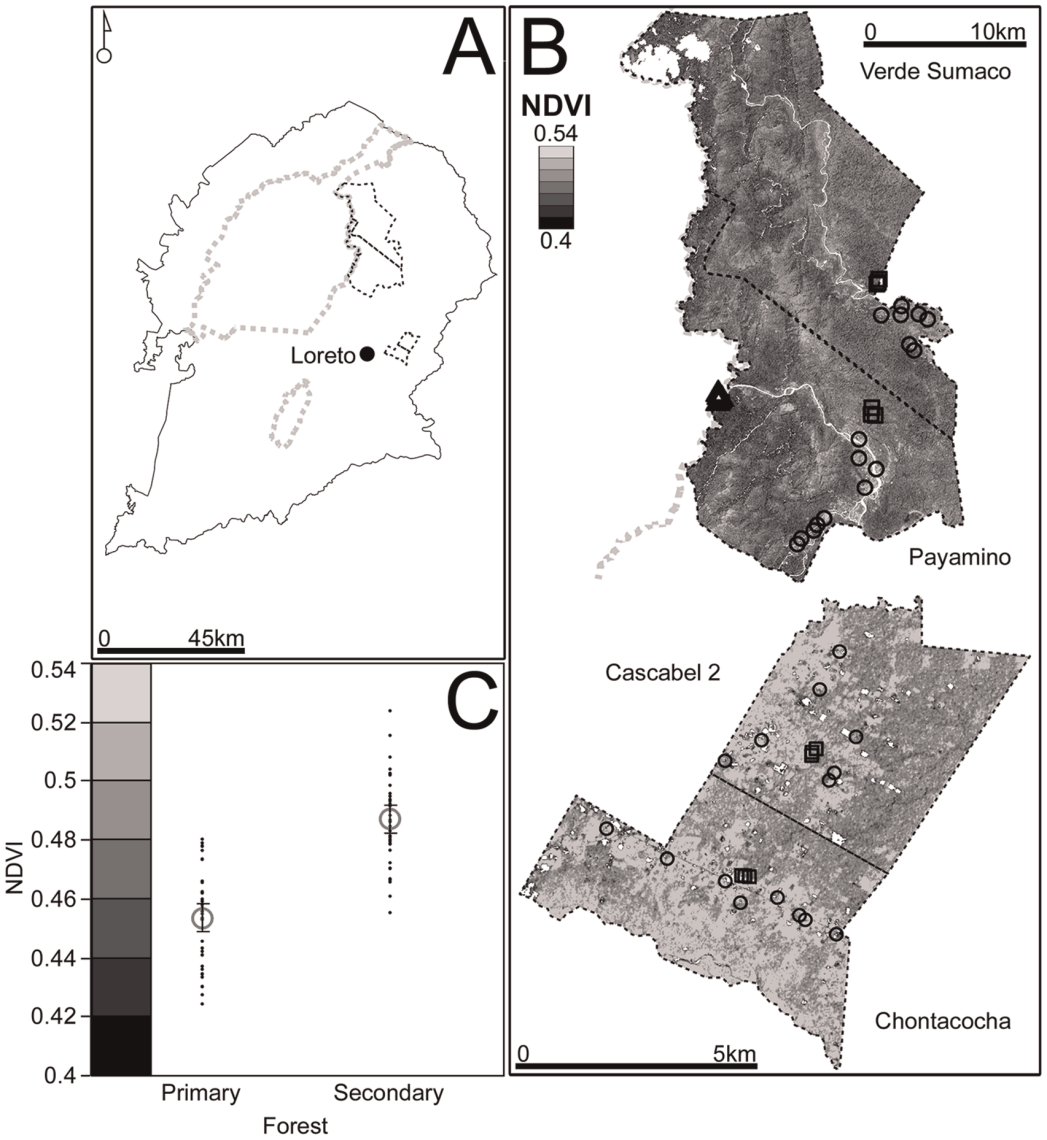
Study sites and Remote Sensing Analysis. A) Sampled communities in the Sumaco Biosphere Reserve. Grey dashed lines represent national park limits and black dashed lines represent community boundaries. B) Study sites and forest cover in remote communities and in non-remote communities. Forest cover is represented using NDVI ranges between 0.4 (black) and 0.54 (light grey). Triangles: National park transects. Squares: Control transects. Circles: Household transects C) Differences in forest cover between primary and secondary forest are and colour scale for NDVI values ranging between 0.4 (black) to 0.54 (light grey). Data are presented as means (grey circles) and individual data points (black dots). Error bars represent 95% confidence intervals.

We conducted our study in the Sumaco Biosphere Reserve (SBR) in the Amazon region of the Ecuadorian tropical Andes; an area classified as a biodiversity hotspot [35]. All necessary permits were obtained for the described field study (Ecuadorian Ministry of the Environment permits numbers: 005-08 IC-FAU-DNBAPVS/MA and 030-2009-FAU-DPO-MA). Sampling took place in four indigenous Kichwa communities within the SBR as well as one site within the Sumaco national park (Fig. 1A, B). The communities included in this study are San José de Payamino (hereafter Payamino), Verde Sumaco and Chontacocha and Cascabel 2 (Fig. 1B). All sampled sites are classified as tropical moist forest [31] and lie at an elevation of approximately 400 m. Sampling took place during the months of July to November in both 2008 (Payamino and Chontacocha) and 2009 (National park, Verde Sumaco, Cascabel 2). The area sampled inside the national park is the only accessible area at 400 m. Although close to the border with Payamino, the area is more than 10 km away from the community’s centre and is inaccessible by road (Fig. 1B).

The community’s populations are very similar and range from 284 to 300 inhabitants in each. Payamino and Verde Sumaco own 17,000 and 24,000 hectares respectively and have little or no access by road. In contrast, Cascabel 2 and Chontacocha own 2,000 hectares each and are easily accessible by road from the nearest market town, Loreto (ca. 10 km).

The national park is an IUCN category II protected area with no history of recent human settlements. In addition to higher population densities and easier access to markets, Cascabel 2 and Chontacocha display agricultural practices typically associated with agricultural intensification and extensification. These include significantly shorter fallow times and larger yields of cash crops as well as significant increases in agricultural and fallow land [36]. We used these socio-economic parameters to classify the remoteness of the sampled communities and classified Payamino and Verde Sumaco as remote and Chontacocha and Cascabel 2 as non-remote.

### Sampling

We sampled epiphytic ferns, leaf litter frogs of the family Strabomantidae (hereafter leaf litter frogs), and dung beetles. Ferns have been shown to be sensitive to disturbance and dependant on the availability of forest refugia for dispersal [28], [37], [38]. Epiphyte diversity increases with forest maturity [39] and decreases with habitat fragmentation [40]. Epiphytic fern diversity is therefore considered a good indicator of forest maturity because it is a result of forest structure and composition and sensitive to disturbance [38]–[41]. Similarly, because their distribution is largely restricted to the forest floor, leaf litter dwelling amphibians are considered accurate indicators of forest health [27], [33], [42]. Dung beetles depend on the nutrients obtained from mammalian dung and are thus thought to be indicative of mammalian populations [43].

We sampled epiphytic ferns, leaf litter frogs and dung beetles along 49, 500 m transects in the national park and in inhabited and uninhabited areas in each community. Difficult terrain, restricted availability of uninhabited areas in non-remote communities and the inaccessibility of the national park and uninhabited areas in remote communities, limited the number of transects we could sample in these sites and prohibited the random placement of transects. While these limitations could exacerbate site-specific effects and need to be considered when drawing conclusion, the sampled sites provided us with the only available basis for comparisons. We sampled five transects in the national park and three transects in uninhabited forest areas in each community. These uninhabited areas served as controls to ensure baseline levels of species richness and were selected to represent areas of least human impact within each community. In remote communities, uninhabited areas were several hours by canoe and foot from the communities’ centre and had no recent history of human settlements. In non-remote communities, uninhabited areas were either a 20 ha forest reserve within short walking distance from the communities’ centre (Chontacocha) or an area within 40 ha of communal forest that had not yet been allocated to community members (Cascabel 2). While neither of the two sites had recent signs of agricultural use, their proximity to the communities’ centers suggests that they are likely to have experienced substantially higher levels of impact (e.g. through selective logging) than uninhabited sites in remote communities. Overall, we sampled 32 transects in forest plots in the proximity of households in the four communities; nine transects each in Payamino and Chontacocha and seven transects each in Verde Sumaco and Cascabel (Fig. 1B). Selection of these sites was dependant on prior consent from community residents. To ensure as representative a sample as possible, we included households involved in both subsistence agriculture, and spread (rather than clustered) over the communities’ inhabited areas.

We cut transects on the first day of sampling. For transects in the proximity of households, we used community residents’ houses points of origins for each transect. With the exception of two cases in non-remote communities, households were never closer than 350 m. Transects were cut in semi-random direction avoiding agricultural plots, rivers, paths and roads whenever possible. We cut transects in uninhabited forest areas in perpendicular directions and transects were never closer than 100 m from each other. In the national park, we cut transects in perpendicular directions and transects where never closer than 250 m.

We measured epiphytic fern richness in ten 5 m×5 m geo-referenced (Gecko 201 GPS unit, Garmin Ltd.) quadrats set out every 50 m along each transect. To minimize fern sampling bias, we set out quadrats parallel to each transect and used 50 m marks as central points. We only included ferns within reach-height (2 m) and within the projected quadrat boundaries. We placed dung-baited pitfall traps at the centre of each quadrat and collected the contents after 24 hr. We measured leaf litter frog species richness on the second day of sampling and standardized the search effort by walking transects at a rate of 50 m per person per hour for a total of 10 person hours for the entire transect and by only searching within one meter distance from either side of the transect midline. Sampling was carried out by a group of four people and the time adjusted to maintain a sampling intensity of 10 person hours when only three were available. We sampled by turning through the leaf litter during the day and searching the understory vegetation during the night. Incidental observations (e.g. if a frog was seen perched on a leaf during the day) were always included. We minimized potential observer biases, by ensuring that the same team of core researchers (JAO, NKT, NT) conducted all sampling efforts on all transects. We identified all epiphytic ferns and leaf litter frogs using field guides [31], [32], [42] and deposited fern reference collections and leaf litter frog reference photographs at the Museo Ecuatoriano de Ciencias Naturales (MECN). We took dung beetles to the MECN for identification.

### Quantification of Secondary Forest and Canopy Cover Analysis

To assess forest condition and identify areas of primary and secondary forest in remote and non-remote communities, we calculated Normalized Difference Vegetation Index (NDVI) values for a single geometrically and atmospherically corrected 15 m resolution ASTER satellite image taken in 2007 (L1B-0030129-2007153753-20171, *RMSE*  = 9.52 pixels). The image covers the four communities and the sampled area inside the national park. We extracted NDVI values for each geo-referenced quadrat and for an additional 40 GPS points recorded in July 2011 in secondary forests aged approximately 5, 10, 20 and 25 years (ten in each forest age category). These additional areas were identified using oral histories of past agricultural and forest use as well as rapid qualitative assessments of forest age and structure, which were performed with the help of local community residents with a good knowledge of local forests. The accuracy of GPS measurements under canopy cover typically ranged between ±8–24 m and we, therefore, averaged pixel values using the eight neighboring pixels for the analysis. Because NDVI values corresponding to the four secondary forest age groups did not differ statistically, we pooled and compared them to the values extracted from the quadrats in the national park, which we classified as primary forest. We then used NDVI value ranges for both primary and secondary forest to produce reclassified maps of remote and non-remote communities (See Fig. 1C). These analyses were performed in ArcGIS 10 (ESRI).

We quantified forest canopy cover by taking digital photographs of the canopy in each quadrat along each transects. We took photographs 50 cm above the ground using a fisheye lens (16 mm f/2.8 AF Nikkor Fisheye lens on a Nikon D300 camera body) and measured canopy cover using specialized canopy gap fraction analysis software [44].

### Analysis

We measured species richness as the total number of species of epiphytic ferns, leaf litter frog species richness and dung beetle along each transect. NDVI and gap fraction values were averaged for each transect. In addition to measuring changes in total richness, we measured how the 25% commonest and rarest species differed along the human induced disturbance gradient.

We measured the relative contribution of common and rare subsets to total species diversity patterns using the methods published by Lennon et al. [45], Pearman and Weber [23] and Mazaris et al. [46]. Briefly, we ranked taxa from commonest to rarest using the relative abundances of species and genera within each taxonomic group. Because not all species were present in all sites, we calculated commonness and rarity separately for each taxon in each site. Next, we created a series of subsets equal to the total number of species or genera. We created each subset by successively including species in ranked order, from commonest to rarest (CtoR) and from rarest to commonest (RtoC). The first subset only contained the commonest (or rarest) species, the second subset contained the first and second most common (or rare) species, and so forth. We plotted correlations between subsets and total species richness for each site and determined the approximation of subsets to total species richness patterns visually.

Finally, we measured the association between estimates of surrogate taxa to determine the presence of each other. As well as measuring the associations of total richness between taxa, we measured the correlation between the 25% most common species and the 25% rarest species in all surrogate taxa. We measured associations using Pearson’s correlations and controlled for geographic distances by using partial Mantel’s tests with geographical distances as a constant. To control for co-linearity, we removed the corresponding common or rare subset from total richness when performing correlations between individual subsets and total richness within taxonomic groups.

To ensure adequate species richness sampling, we used Chao 1, Jackknife 1 and Jackknife 2 to estimate total leaf litter frog and dung beetles species richness and used Chao 2, Jackknife 1 and Jackknife 2 to estimate epiphytic fern species richness. These estimators are commonly used for calculating total species richness using both abundance and presence/absence data [47], [48].

We used three separate approaches to investigate the relationships between remoteness, species richness and measures of forest cover. Firstly, we performed a series of linear and first polynomial quadratic regressions to look for relationships between taxonomic groups and NDVI and canopy cover, presenting the results showing the strongest relationships. Secondly, we analyzed between site differences in species richness, NDVI and canopy cover using single predictor Generalized Linear Models (GLMs) to control for unequal variances in the data, Finally, we combined effects of both site and NDVI on species richness by performing a series of full factorial GLMs with both site and NDVI as predictor variables. We analyzed individual differences between the national park, remote and non-remote sites using single degree of freedom contrast tests (SDF) and performed Bonferroni corrections to control for the number of individual tests. Canopy cover data were not normally distributed and we log transformed them for the analysis. We performed regressions, correlations and GLMs were in JMP 8 (SAS Institute Inc) used PASSaGE 2.0 to run Mantel test’s [49].

## Results

### NDVI, Canopy Cover and Taxonomic Richness

**Figure 2:**
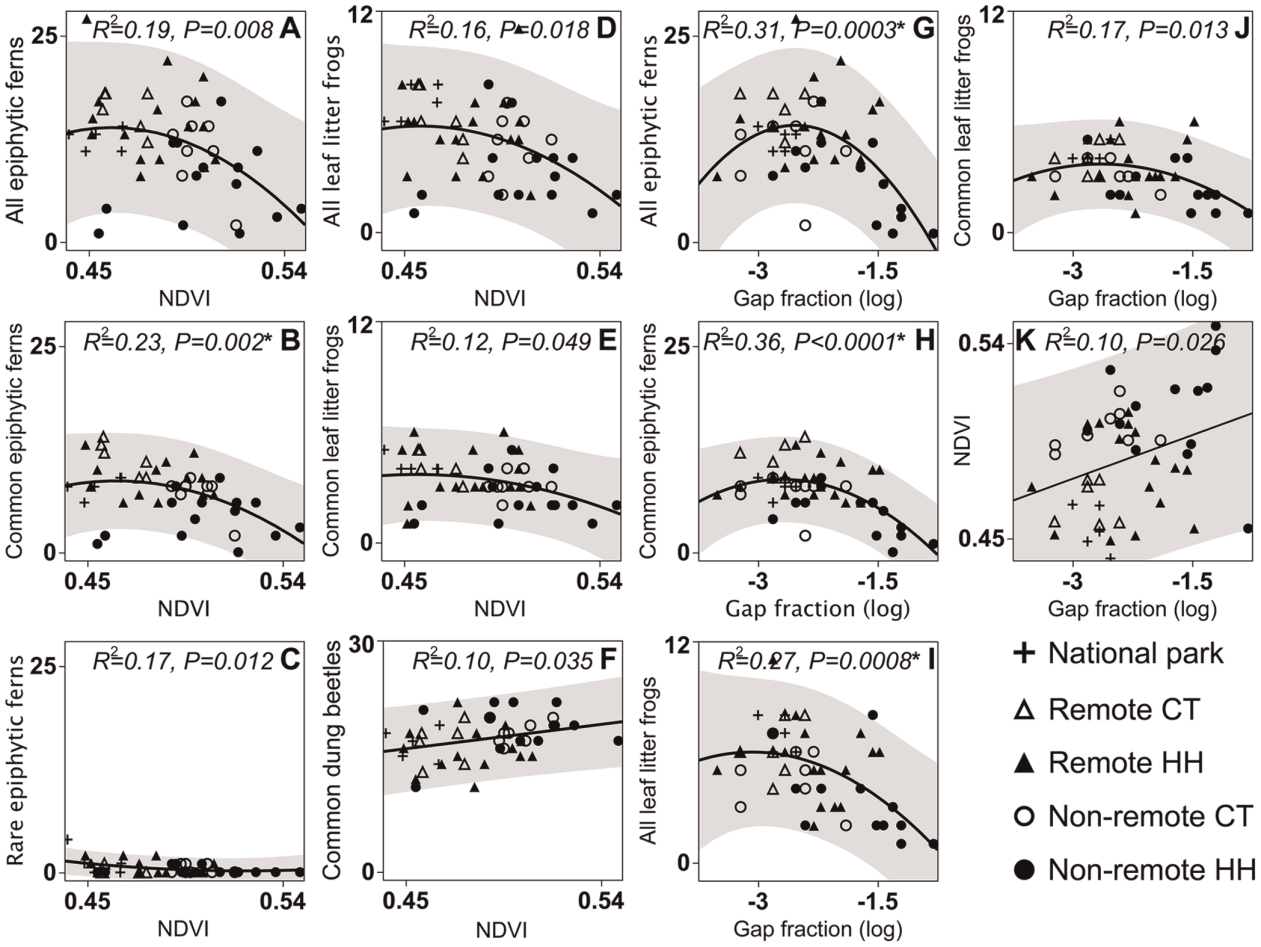
Significant regressions between NDVI, canopy cover (gap fraction) and taxonomic richness. A–C) NDVI and total, common and rare epiphytic fern species richness. D, E) NDVI and total and common leaf litter frog species richness. F) NDVI and common dung beetle species richness. G, H) Gap fraction and total and common epiphytic fern species richness. I, J) Gap fraction and total and common leaf litter frog species richness. K) Gap fraction and NDVI. Lines denote regression fits and gray shading denotes 95% Confidence intervals. Asterisks denote regression significant after Bonferroni corrections. CT = Control transects, HH = Household transects.

Overall total, common and rare epiphytic fern species, total and common leaf litter frogs and common beetle species were negatively related to NDVI (Fig. 2), yet only the relationship between NDVI and common epiphytic fern species remained significant after Bonferroni correction (Fig. 2B). Similarly, total and common epiphytic fern species, total and common leaf litter frog species decreased with canopy gap fraction (Fig. 2) yet only the relationships between gap fraction and total (Fig. 2G) and common (Fig. 2H) epiphytic fern species richness and total leaf litter frog species richness (Fig. 2I) remained significant after Bonferroni correction. NDVI increased with gap fraction but regression results were not significant after Bonferroni correction (Fig. 2K).

**Figure 3:**
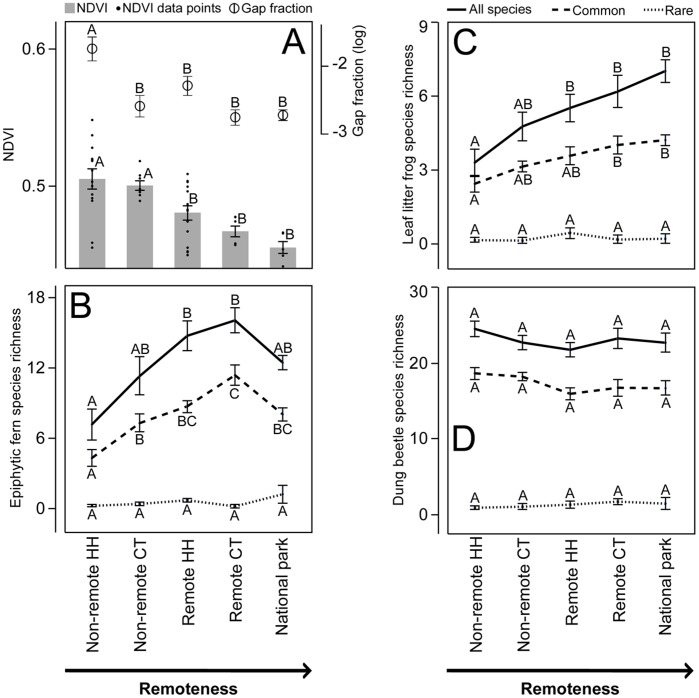
Differences in NDVI, canopy cover and species richness. A) Differences in NDVI and canopy cover (gap fraction). B) Differences in epiphytic fern species. C) Differences in leaf litter frogs. D) Differences in dung beetles. Solid lines represent all species while dashed and dotter lines represent the 25% commonest and 25% rarest subsets. With the exception of NDVI in A), which also includes individual data points, data are presented as means with error bars representing one standard error. Points not connected by the same letter denote single degree of freedom contrast tests that differ significantly after Bonferroni correction. HH = Household transects, CT = Control transects.

NDVI values in primary forest were significantly lower than values in secondary forest (P>0.0001, t = 10.01, df = 1, Fig. 1C) and decreased significantly with remoteness despite substantial variation in household transects (P<0.0001, **χ^2^** = 29.22, df = 4, Fig. 3A). Reclassified maps showed substantially higher levels secondary forest and habitat fragmentation in non-remote communities (Fig. 1B). Similarly, canopy gap fraction decreased with remoteness (P = 0.0002, **χ^2^** = 22.61, df = 4, Fig. 3A) with canopy cover being significantly lower in non-remote household forest plots than in the national park, remote and non-remote control transects. Canopy cover in remote control transects was also significantly lower than in remote household forest plots.

We sampled 1,424 epiphytic ferns, 598 leaf litter frogs and 14,690 dung beetles and found 84 species of epiphytic ferns, 28 species of leaf litter frogs and 93 species of dung beetles. Accumulation curves for all there taxa did not level off completely (See Appendix S1). Chao 2, Jackknife 1 and Jackknife 2 richness estimates for epiphytic ferns yielded total richness estimates of 115, 104 and 118, suggesting that we sampled between 70% and 80% of all epiphytic fern species found in our sample sites. Chao 1, Jackknife 1 and Jackknife 2 richness estimates for leaf litter frogs yielded species richness estimates of 29, 30 and 29 respectively, suggesting that we sampled between 90% and 99% of all leaf litter frogs species present in our sample sites. Chao 1, Jackknife 1 and Jackknife 2 for beetle genera richness yielded total species richness estimates of 112, 103 and 108 suggesting that we sampled between 83% and 91% of the total beetle species richness in our sample sites.

Total epiphytic fern species richness increased with remoteness, peaking at remote control sites before decreasing slightly in the national park (Fig. 3B). These difference between sites were significant according to the single predictor GLM (P = 0.0001, **χ^2^** = 23.51, df = 4) as well as the full factorial GLM (P<0.0001, **χ^2^** = 28.77, df = 9), the latter showing site as being the only significant effect variable in the model (P = 0.0012, **χ^2^** = 18.05, df = 4). Although total epiphytic fern species richness did not vary significantly between the national park, remote community sites and non-remote control transects (Fig. 3B), richness in non-remote household transects was significantly lower than in remote household (P<0.0001, **χ^2^** = 19.17, df = 1) and control transects (P<0.0001, **χ^2^** = 15.46, df = 1).

Common epiphytic fern species richness also increased with remoteness and followed a similar pattern to total epiphytic fern species richness, with richness peaking in remote control sites before decreasing slightly in the national park (Fig. 3B). These difference between sites were significant according to the single predictor GLM (P<0.0001, **χ^2^** = 37.78, df = 4) as well as the full factorial GLM (P<0.0001, **χ^2^** = 46.56, df = 9), the latter showing site as being the only significant effect variable in the model (P = 0.0012, **χ^2^** = 18.05, df = 4). Although common epiphytic fern species richness did not vary between the national park, remote sites and non-remote control transects (Fig. 3B), richness in non-remote household transects was significantly lower than in non-remote control sites (P<0.0026, **χ^2^** = 9.03, df = 1), remote household (P<0.0001, **χ^2^** = 24.66, df = 1) and control (P<0.0001, **χ^2^** = 32.55, df = 1) transects and the national park (P<0.0014, **χ^2^** = 10.13, df = 1).

Rare epiphytic species richness only differed between sites using the full factorial GLM did not vary between sites according to the single predictor GLM results (P<0.002, **χ^2^** = 25.86, df = 9) with site (P = 0.037, **χ^2^** = 10.21, df = 4), NDVI (P = 0.0014, **χ^2^** = 10.26, df = 1) and the interaction between them being significant (P = 0.013, **χ^2^** = 12.57, df = 4).

Total leaf litter frog species richness increased with remoteness and was highest in the national park (Fig. 3C). Differences between sites were significant according to the single predictor GLM (P = 0.0013, **χ^2^** = 17.91, df = 4) as well as the full factorial GLM (P  = 0.01, **χ^2^** = 21.06, df = 9) with site being the only significant effect variable in the model (P = 0.035, **χ^2^** = 10.34, df = 4). Although leaf litter frog species richness did not differ between the national park, remote sites and non-remote control transects (Fig. 3C), species in non-remote household transects was significantly lower than in the national park (P = 0.0003, **χ^2^** = 13.01, df = 1), and remote control (P = 0.0023, **χ^2^** = 9.28, df = 1) and household (P = 0.0018, **χ^2^** = 9.70, df = 1) transects.

Common leaf litter frog species richness also increased with remoteness and followed a similar pattern to total species richness, with richness peaking in the national park (Fig. 3C). Differences between sites were significant according to the single predictor GLM (P = 0.0085, **χ^2^** = 13.64, df = 4) and while species richness did not differ between the national park, remote sites and non-remote control sites (Fig. 3C), species richness in non-remote household transects was significantly lower than in remote control transects (P = 0.0048, **χ^2^** = 7.92, df = 1) and the national park (P = 0.0031, **χ^2^** = 8.76, df = 1). The full factorial model was not significant.

Neither of the GLM models showed any differences for rare leaf litter frog species richness between sites. Similarly, neither of the GLM models showed any significant differences in total, common and rare dung beetle species richness between sites (Fig. 3D).

### Contribution of Common and Rare Species Richness to Overall Species Richness

**Figure 4:**
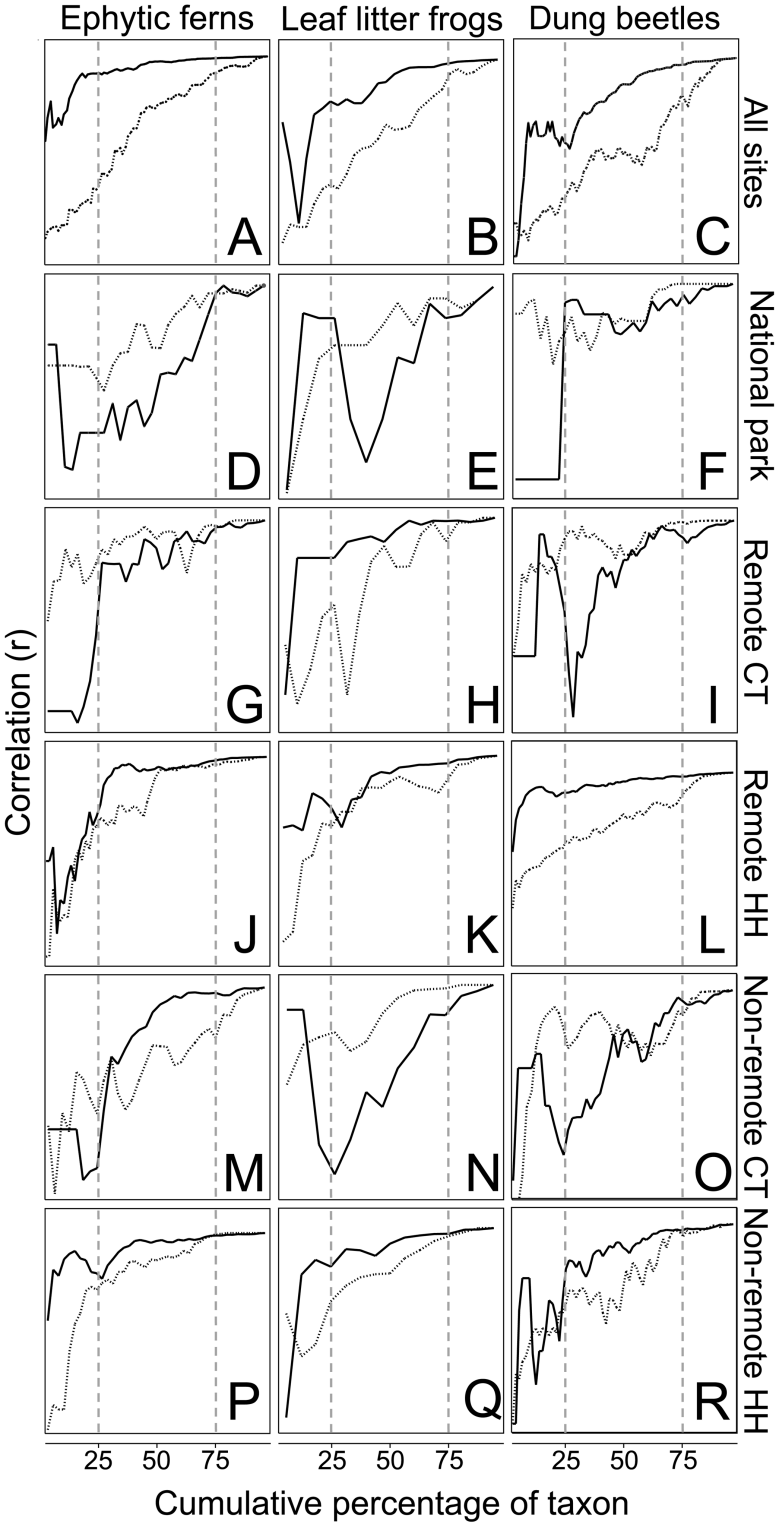
Contribution of common and rare species to general patterns of species richness. Solid lines: Commonest species. Dashed lines: Rarest species. Subsets of species were constructed by ranking species according to relative abundances from common to rare or rare to common. Subsets were then successively correlated to overall species richness. CT = Control transects, HH = Household transects.

The contribution of common and rare species to overall species richness differed between taxonomic groups and between sites (Fig. 4). Across all sites, the CtoR assembly of all taxa showed higher correlations to overall richness than the RtoC assemblies (Fig. 4A–C). Conversely, in the national park, RtoC assemblies of all taxa showed higher correlations to overall species richness than CtoR assemblies (Fig. 4D–F). In remote control transects, RtoC assemblies of epiphytic fern (Fig. 4G) and dung beetles (Fig. 4I) species had higher correlations to overall species richness than CtoR assemblies, whereas CtoR assemblies of leaf litter frogs showed higher correlations to overall species richness than RtoC assemblies (Fig. 4H). In remote household transects, CtoR assemblies of all taxa showed higher correlations to overall species richness RtoC (Fig. 4J–L). In non-remote control transects, RtoC assemblies of leaf litter frogs (Fig. 4N) and dung beetles (Fig. 4O) showed higher correlations to overall species richness than CtoR assemblies, whereas CtoR of epiphytic ferns showed higher correlations to overall species richness than RtoC (Fig. 4M). In non-remote household transects, the CtoR assembly of all taxa showed higher correlations to overall richness than the RtoC assemblies (Fig. 4P–R).

### Relationships between Taxa

Pearson’s correlations and Mantel’s tests results show strong significant positive relationships between total epiphytic fern species and total leaf litter frog species richness and between common epiphytic fern species and common leaf litter frog species (Table 1). Associations between common beetle and rare leaf litter frog species richness were not significant when analyzed using Pearson’s correlations but were significant when analyzed using Mantel’s test (Table 1).

**Table 1:** Correlation results between rare and common species.

		Pearson’s r	P	Mantel’s r	P
All Species	Ferns v. frogs	0.51	0.0002*	0.41	<0.0001*
	Ferns v. beetles	−0.17	0.24	−0.01	0.89
	Beetles v. frogs	0.08	0.58	0.07	0.44
Common Species	Ferns v. frogs	0.59	<0.0001*	0.37	<.0001*
	Ferns v. beetles	−0.30	0.045	0.01	0.91
	Beetles v. frogs	−0.09	0.21	0.04	0.64
Rare Species	Ferns v. frogs	<0.01	0.97	−0.02	0.79
	Ferns v. beetles	0.35	0.018	<0.01	0.95
	Beetles v. frogs	−0.13	0.39	−0.02	0.59
Common ferns v rare species	v. rare ferns	0.17	0.25	−0.1	0.18
	v. rare frogs	0.02	0.90	0.01	0.92
	v. rare beetles	0.27	0.06	−0.02	0.67
Common frogs v. rare species	v. rare frogs	0.07	0.63	−0.08	0.34
	v. rare ferns	0.21	0.15	−0.02	0.78
	v. rare beetles	0.20	0.16	−0.06	0.21
Common beetles v rare species	v. rare beetles	−0.07	0.61	0.01	0.84
	v. rare ferns	−0.18	0.21	0.05	0.52
	v. rare frogs	0.09	0.56	0.42	0.0003*

* denotes significance after Bonferroni correction.

Pearson’s correlations and Mantel’s test results show positive significant associations between common epiphytic ferns species and total epiphytic fern species richness as well as between common epiphytic ferns species and total leaf litter frog species richness (Table 2). Similarly, Pearson’s correlation and Mantel’s test results for common leaf litter frog and total epiphytic fern species were also significant.

**Table 2:** Correlation between total species richness, and rare and common species.

	Pearson’sr	P	Mantel’sr	Mantel’sP
common ferns v. all ferns	0.40	<0.0001*	0.53	<0.0001*
rare ferns v. all ferns	0.09	0.51	−0.09	0.22
common ferns v. all frogs	0.54	<0.0001*	0.36	<0.0001*
rare ferns v. all frogs	0.17	0.24	−0.07	0.26
common ferns v. all beetles	−0.15	0.29	−0.04	0.72
rare ferns v. all beetles	0.10	0.51	−0.02	0.72
common frogs v. all frogs	0.35	0.013	0.13	0.006
rare frogs v. all frogs	−0.13	0.28	−0.03	0.63
common frogs v. all ferns	0.57	<0.0001*	0.38	<0.0001*
rare frogs v. all ferns	0.02	0.89	−0.04	0.41
common frogs v. all beetles	−0.02	0.87	0.02	0.98
rare frogs v. all beetles	0.08	0.58	0.25	0.03
common beetles v.all beetles	−0.08	0.62	0.33	0.003
rare beetles v. all beetles	−0.25	0.089	−0.06	0.29
common beetles v. all ferns	−0.34	0.02	<0.01	0.96
rare beetles v. all ferns	0.23	0.13	−0.04	0.41
common beetles v. all frogs	0.04	0.79	0.13	0.15
rare beetles v. all frogs	0.25	0.09	−0.04	0.29

* denotes significance after Bonferroni correction.

## Discussion

Our results suggest that environmental degradation is likely to affect common species rather than rare ones (See Fig. 2 and 3). Like others before us [13]–[16], we show that despite relatively complex patterns of species co-occurrence, organisms have the potential to act as reliable indicators of diversity of forest condition (Fig. 2) and other taxonomic groups or species (Tables 1 & 2). These relationships, however, are likely to be due to common species rather than rare ones. We also provide evidence that human disturbance gradients differentially affect common and rare species and that the changes in common species affect how common and rare species contribute to overall richness patterns. This is of particular importance for the use of indicator taxa and estimations of conservation value, because, although rare species can respond to environmental changes, they have been previously shown to be poor predictors of broader biological changes [5]–[7].

### Taxonomic Richness

NDVI values are negatively correlated with tropical forest age and canopy complexity [50], suggesting that our measures of remoteness correctly reflected environmental degradation. While our measures of forest cover were significant predictors of taxonomic richness, site remoteness was the best predictor of epiphytic fern and leaf litter frog species richness. NDVI measures in household transects varied significantly, suggesting possible site-specific interaction effects, which could explain why the full factorial GLM for rare epiphytic ferns was significant and why, overall, site remoteness was a better predictor of taxonomic richness.

Changes in forest structure and composition affect species richness patterns through species dispersal mechanisms, the availability of light and soil nutrient cycling [41], [51]. The observed changes in epiphytic fern and leaf litter frog species richness are likely to be caused by changes in forest condition, canopy cover, increases in secondary forest and habitat fragmentation in non-remote communities. While canopy cover was lower in inhabited areas, there is evidence that hemispherical photography based measures of canopy cover cannot differentiate between secondary and primary forest [52]. The inclusion of additional measures of forest structure (e.g. diameter at breast height of all trees above a certain diameter within sampled quadrats) could provide further links between species diversity and forest condition [33].

Higher epiphytic fern species richness in remote sites, which explain much of the statistical difference between these sites and inhabited remote areas, could be related to intermediate levels of disturbance [8], [53], [54] and be mediated through a combination of increases in availability of light in the understory, which can widen epiphytic niches [55], and increases in the abundance of small understory trees, which have been shown to provide good habitats for epiphytes in other regions of the tropical Andes [56]. While remote areas appear to be somewhat more diverse than the national park, these differences are not statistically significant. The inaccessibility and difficult terrain of the national park meant that we were unable to sample other sites at the same elevation. It is, therefore, difficult to conclusively differentiate between patch specific effects and real ecological patterns. Furthermore, we only sampled epiphytic ferns within reach height. Although ferns are one of the dominant epiphyte groups inhabiting the lower sections of tree-trunks [57], [58], they also contribute to epiphyte diversity in higher regions of the forest understory [58]. Limiting our sampling to ferns within reach height could help explain why we only sampled 70–80% of the estimated epiphytic fern species present in our sites.

Although dung beetles have been considered reliable indicators of forest health [43], [59] there is evidence that they might not be responsive to differences between certain forest types [27], [60]. Agricultural changes and the inclusion of livestock are known to change dung beetle species composition [61]. Although our results suggest that beetle species richness might not be a sufficiently sensitive indicator of human disturbance it is possible that a more detailed analysis of changes in particular functional groups could yield different results.

### Correspondence of Common and Rare Species Richness to Total Species Richness

Rare species are usually more speciose but have lower relative abundances than common species [34]. Consequently, common species richness patterns are thought to resemble overall richness patterns more closely than rare species richness patterns [23], [45], [46]. If, however, the variance between individual data points is large and the commonest species are ubiquitous - with little variance between points, then overall richness will correlate better with rarer subsets because these will account for most of the variation between data points [45].

Human disturbance can change species composition [62], [63]. The different contributions of common and rare species to overall richness patterns and the changes of common epiphytic fern and leaf litter frog species in our data, suggest that human disturbance is likely to have changed the contribution of common species by affecting the dominance patterns of common species and increasing the variance between data points.

This analysis (see also [23], [45], [46]), however, relies on presence/absence data. Common and rare species were weighted equally and this effectively reduces the variation between data points. Furthermore, some of our analyses relied on relatively small sample sizes (national park and uninhabited control sites), which might affect variances between data points and influence the contribution of common and rare species to overall richness patterns. Larger datasets using species relative abundances could provide more detailed information about how common and rare species subsets affect overall patterns of species diversity.

Our epiphytic fern and leaf litter frog data suggest that measuring common species in inhabited areas and rare species in uninhabited areas might provide accurate information on overall richness patterns. This, however, might be difficult to implement in practice since the relative commonness and rarity of species can only be assessed by identifying all species within a sample. Furthermore, species composition is likely to vary over time and space and the relationship between common and rare species must be constantly asserted suggesting that in addition to any practical considerations, monitoring efforts solely focusing on either common or rare species are likely to provide unreliable information on species richness patterns.

### Relationships between Taxa

Coarse-scale studies have shown that the presence of rare or threatened species does not necessarily overlap with areas of high species diversity [5]–[7]. Since only total or common richness patterns were consistently related to each other and/or measures of forest degradation, our results show that a similar pattern also occurs at a smaller scale. These results have potential implications for monitoring initiatives focusing on species threat. Since rarity often increases with threat, monitoring efforts focusing on species threat might fail to provide reliable information on overall richness patterns of other taxa.

Our results suggest that leaf litter frogs and epiphytic ferns might be good surrogate measures of environmental degradation and each other in Ecuador [33] and, perhaps, other Neotropical regions [27], [28], [60], [64]. Their broader potential as indicators, however, relies on more comprehensive studies linking their relationship to additional measures of forest structure and composition in other geographical regions as well as their co-occurrence patterns with other taxonomic groups (e.g. birds or mammals) with varying habitat requirements.

### Conclusion

In addition to providing further evidence that patterns of co-occurrence largely rely on the particular species in question [27], [28], [60], our results show that environmental degradation is likely to differentially affect the relationship between common and rare species within and between taxa. Studies comparing species compositions and patterns of co-abundance across environmental gradients are particularly useful for the development of monitoring tools because habitat changes can help highlight patterns of co-occurence [23]. While the literature on biodiversity indicators is extensive, standardized, comprehensive and comparative studies are rare. In order to better understand how patterns of co-occurrence respond to environmental changes, there is a need for a more coordinated approach to assess how anthropogenic factors influence the relationship between different organisms, across differing gradients and at various scales.

Conservation policy decisions based on limited data from a few taxa will undoubtedly remain questionable and comprehensive studies aiming to maximise biological relevancy under economical constraints should consider monitoring as many taxa as financially possible [65]. In addition to potential economic constraints, however, multi-taxon assessments often require a significant degree of coordination between teams of experts. If easily identifiable and representative taxa can provide rapid initial assessment tools to help identify priority areas for further consideration with less money and without the need for large teams of experts, e.g. through more locally-based monitoring initiatives [66], then their role is essential as we struggle to track environmental changes with limited time, money and expertise.

## Supporting Information

Appendix S1 Species saturation curves for A) epiphytic ferns, B) leaf litter frogs and C) dung beetles. Open circles represent individual data points. Black lines represent quadratic polynomial lines of best fit. Shaded areas represent 95% confidence intervals.
(TIF)
