# Supplementary figures and images for: Co-Occurrence Patterns of Common and Rare Leaf-Litter Frogs, Epiphytic Ferns and Dung Beetles across a Gradient of Human Disturbance
Source: PLoS One. 2012 Jun 11;7(6):e38922. doi: 10.1371/journal.pone.0038922 (PMC3372485; doi:10.1371/journal.pone.0038922)

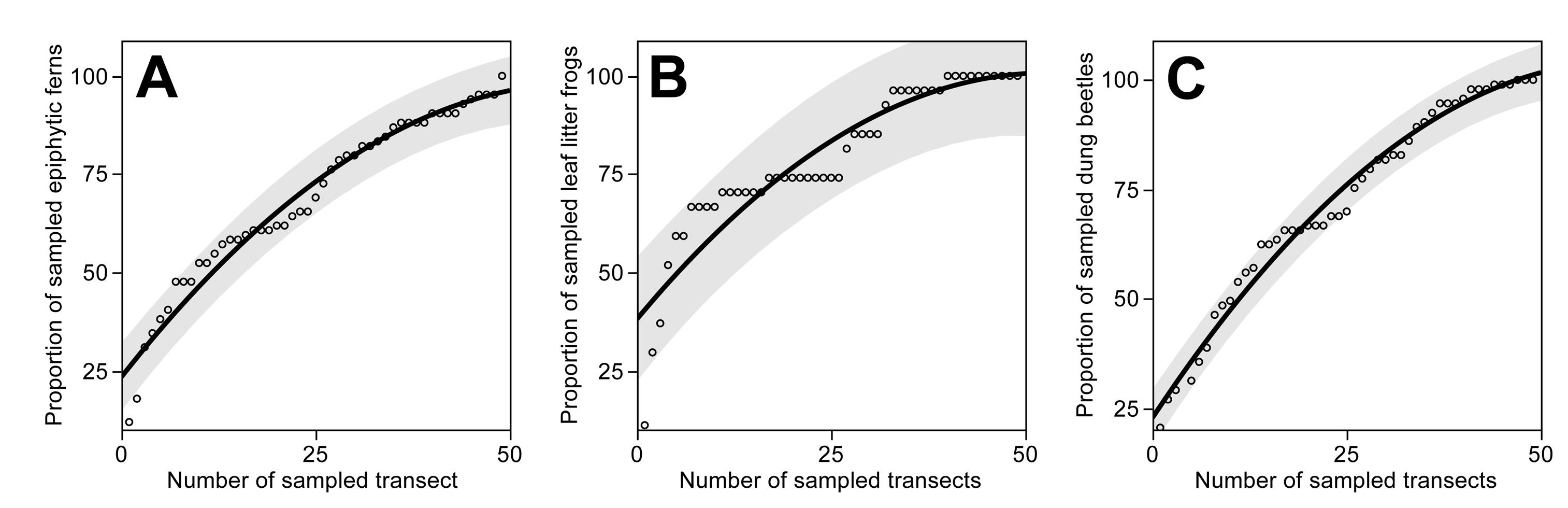

Supplement: Appendix S1 — Species saturation curves for A) epiphytic ferns, B) leaf litter frogs and C) dung beetles. Open circles represent individual data points. Black lines represent quadratic polynomial lines of best fit. Shaded areas represent 95% confidence intervals. (TIF) [file pone.0038922.s001.tif]
